# Address of the President of the Medical Society of the County of Erie, Dec. 18, 1916

**Published:** 1917-03

**Authors:** Franklin W. Barrows

**Affiliations:** Buffalo


### Address of the President of the Medical Society of the County of Erie, Dec. 18, 1916.

DR. FRANKLIN W. BARROWS, Buffalo.

The experiences of one year as president of the County Medical Society have convinced the speaker that he is just beginning to learn the possibilities of the office. It is only natural for him to fancy that if he could serve the society another year he would be able to avoid some mistakes and to perform his duties more efficiently. But custom very wisely limits the presidency to one term and relegates the retiring officer to the ranks. All his ambitions for the future of the society become the common property of the membership. As a past president he will always have a keener interest than ever before in the affairs of the society and a larger conception of the dignity and power of the organization. The speaker acknowledges his hearty appreciation of the support that he always received from the officers and members of the society. The gratifying growth of our membership and the large attendance at our meetings have given evidence of the loyalty of the medical profession to the one organization that is most vitally concerned with their professional and economic welfare. Your enthusiastic reception of the president of our state society and his associates at our last meeting was most encouraging to them and highly creditable to yourselves. For all these expressions of your fraternity and your interest in the ideals of our state and county organizations I thank you once again.

It is a pleasure to report that your Council has been faithful to its duties and that its meetings have been marked by a spirit of unity and keen interest in the objects of the society. Our thanks are due to them individually and as a body for their sacrifice of time and their thoughtful transaction of the business committed to them. In view of the responsible duties to the Council your president would recommend that our by-laws be so amended that the retiring president shall become a member of the council during the term of office of his successor, this rule to become effective one year from today.

Your president regrets that during the past year the members of our society residing outside of Buffalo have had little or no part in our meetings although they are as much members as the rest of us and have contributed their part to our support. He would respectfully recommend that next year we hold one of our meetings outside of the city and promote the good fellowship that ought to be as wide as the county.

Following the suggestions of President Tinker, of the State

Society, the Council undertook a canvass of the candidates for the State Senate and Assembly just before our last election, in order to ascertain their standing on matters of vital interest to the medical profession. Unfortunately this canvass was begun but a few days before election and the number of responses was too small to be of service to our members on election day. Your president would strongly recommend that hereafter at the beginning of every state campaign the candidates for Senate and Assembly be asked to give their pledges in support of the legislative measures on matters of public health which commend themselves to the members of our society, and that our members be fully informed on the standing of every candidate before election.

Within the past decade we have all noted an enormous development of public health activities all over the United States. At the same time legislation in many of our states has undertaken to regulate workmen's compensation, and more ambitious plans are openly debated which, in time, bid fair to socialize the practice of medicine as has been done to so great an extent in some other countries. It behooves the medical profession to be on the alert. Changes are bound to occur within a short time which may affect very vitally the relations of the physician to the public. If the profession as a whole is not ready to take a hand in these revolutionary enactments, the legislatures will impose laws upon them which may not be to their liking. It should be the duty of every organization of physicians to study carefully the tendencies along right lines. To this end your president would recommend that some action be taken by this society looking toward the education of our members on these questions in order that we may all be prepared to maintain that position that will be most advantageous to the profession and to the health of those for whom the profession is responsible.

Follow Dr. Tinker's advice, and give the younger members of our society some definite part to perform which will make them feel a greater responsibility for the progress of the profession.

---

**Bacteriaemias in the Agonal Period.** J. W. Fredette, Pittsburg, Jour. of Lab. & Clin. Med., Dec. 1916. In 119 cases, 42 showed positive cultures, 31 of a single, 11 of 2 organisms. The latter are: streptococcus salivarius, pneumococcus, streptococcus pyogenes, staphylococcus pyogenes, aureus, B. typhosus, streptococcus viridans, streptococcus mitis, B. acidi lactici, streptococcus anginosus, streptococcus mucosus, staphylococcus albus, streptococcus haemolyticus, streptococcus infrequens.

